# Galectin-8A Inhibits Cry11Aa Binding to ALP1 and APN 2 Receptors and Toxicity Against *Aedes aegypti* Larvae

**DOI:** 10.3390/toxins17090451

**Published:** 2025-09-06

**Authors:** Xiaohua Hu, Xianhui Huang, Jiannan Liu, Guohui Zhao, Songqing Wu, Xiaoqiang Yu, Lei Xu, Xiong Guan, Lingling Zhang

**Affiliations:** 1 College of Oceanology and Food Science, Quanzhou Normal University, Quanzhou 362000, China; 2 State Key Laboratory of Ecological Pest Control for Fujian and Taiwan Crops, School of Life Science, Fujian Agriculture and Forestry University, Fuzhou 350002, China; 3 Guangdong Provincial Key Laboratory of Insect Developmental Biology and Applied Technology, Institute of Insect Science and Technology, School of Life Sciences, South China Normal University, Guangzhou 510631, China

**Keywords:** *Aedes aegypti*, Bti, Cry11Aa, Galectin-8A, receptor

## Abstract

*Aedes aegypti,* a crucial vector mosquito that transmits many diseases that cause millions of deaths worldwide, can be controlled with *Bacillus thuringiensis* subsp. *israelensis* (Bti). The larvicidal activity of Bti against *Ae. aegypti* is due primarily to Cry4Aa, Cry4Ba, and Cry11Aa, and Cyt1Aa, a protein that synergizes the activity of the Cry proteins. Interestingly, Galectins-6 and Galectins-14, members of a family of β-galactoside-binding proteins that play a role in immune responses insects, have been shown to decrease the activity of Bti toxins. The activity of other Galectins, particularly Galectin-8A, against the Cry proteins is not known. Toward this end, we cloned the gene coding for galactin-8A and expressed the recombinant protein and purified protein. The bioassay results indicated that Galectin-8A can also reduce the toxicity of Cry11Aa, but it was much stronger than Galectin-6. To investigate the interactions among Galectin-8A, Cry11Aa, and toxin receptors, Octet Red System analysis, Western blot, far-Western blot, and ELISA assay were also performed. The Octet Red System result showed that Galectin-8A could also bind to BBMVs of *Ae. aegypti*, with a lower kDa value than that of Galectin-6, indicating that Galectin-8A had a stronger binding affinity to BBMVs than Galectin-6. Western blot, far-Western blot, and ELISA assay analyses also demonstrated that Galectin-8A bound to *Ae. aegypti* receptor ALP1 and APN2, consistent with the protein docking simulation results. These findings support the conclusion that Galectin-8A blocks with ALP1 and APN2 more effectively than Galectin-6, which may subsequently reduce the toxicity of Cry11Aa in *Ae. aegypti*.

## 1. Introduction

Dengue fever is a type of viral disease transmitted by mosquitoes that has become a global public health concern due to the global increase in the spread of the disease in recent decades [1,2,3]. *Ae. aegypti* is the main vector for the transmission of dengue virus, yellow fever virus, and chikungunya [4,5]. Thus, controlling *Ae. aegypti* population is crucial for reducing the burden of arboviral infections. Traditionally, mosquito control depended on chemical insecticides, which proved successful initially, but their overuse has led to environmental pollution and the development of resistance [6,7]. Recently, biological pesticides have been widely applied and promoted as being an environmentally friendly approach with high specificity, exerting little influence on non-target organisms and reducing insect resistance [8,9]. Thus, biological pesticides have been regarded as an ideal way to control mosquitoes. *Bacillus thuringiensis* is a biological pesticide that has been widely used in pest management in agriculture, forestry, and public health [10,11]. *Bt subspecies israelensis* (Bti), which produces crystal toxin protein inclusions during sporulation that are composed of different Cry toxin proteins, could kill mosquitoes, including Cry4Aa, Cry4Ba, Cry10, Cry11Aa, and Cyt1Aa [11,12,13].

Bt Cry toxin proteins are characterized by three structurally conserved domains, including domain I, domain II, and domain III, which hold specific functional roles [14]. Domain I is responsible for the conformational changes in the cell membrane surface, thereby causing the Cry toxin insertion into the midgut cells and leading to perforation. Domain II is mainly composed of a β-prism structure, which recognizes the anchored glucosyl groups on the glycoprotein that directly affects the binding of Cry toxin to different receptors in the insects. And domain III plays an important role in determining the specificity of Cry toxins to insects [15,16,17,18,19]. Cry toxins interact with specific receptors, resulting in the formation of a pre-pore oligomeric structure that is inserted into the cell membrane and causes cell death [20]. In previous studies, Cry11Aa toxins were bound to receptor proteins, such as aminopeptidase N (APN), alkaline phosphatase (ALP), cadherin, and ATP-binding cassette (ABC) transporters [21,22,23,24,25]. This toxin receptor interaction causes pore formation in the epithelial cells, resulting in osmotic imbalance and cell death. Lectins exhibit structural similarity to Cry toxins; they both exhibit a β-prism structure [21,26,27]. Therefore, lectin could interrupt interaction of Cry toxins with their receptors by binding competitively to the glycosylated receptor.

Lectins are a class of proteins that can bind to specific glycoproteins and are widely distributed in animals, plants, and microorganisms. C-type lectins are a superfamily of proteins found in almost all vertebrates and invertebrates. They play an important role in innate immune defenses, development, and epidermal structure [28]. C-type lectin functions in the insect innate immune system by participating in hemocyte nodule formation, activating prophenol oxidase in hemolymph, and promoting the encapsulation and melanization of cells, and enhances phagocytosis, which is an important part of insect immune regulation [29,30,31]. In the previously reported study, *Ae. aegypti* C-type lectin 20, Galectin-6 and Galectin-14 were also found to modulate the toxicity of Cry11Aa, as they could compete with Cry11Aa for binding to BBMVs, ALP1 and APN2, thereby preventing effective binding of toxin to receptor [21,26,27]. There are several Galectins expressed in the *Ae. aegypti* midgut, which can also modulate Cry toxicity to kill mosquitoes. To further understand the function and molecular mechanism of other galectin genes in Cry toxicity, we expressed Galectin-8A in the midgut of *Ae. aegypti* in this study. We cloned, expressed, and purified the recombinant protein Galectin-8A. Toxin experiments were performed to identify if Galectin-8A could affect Cry toxicity. Protein–protein interactions among Galectin-8A, Cry11Aa, and toxin receptors were tested to investigate the mechanism of Galectin-8A in Bt toxins. In addition, a comparison between Galectin-8A and Galectin-6 in Bt toxin mechanisms may be helpful for implementing new biocontrol strategies involving galectins in Bt toxicity.

## 2. Results

### 2.1. The Recombinant Galectin-8A Protein Purified from Ae. aegypti

Galectins are a family of β-galactoside-binding proteins that are widely distributed in insect species and play an important role in insect immune response. In our previous work, we found that five galectin genes were expressed in the midgut of *Ae. aegypti* [18]. In order to understand the different functional roles of galectins, we cloned, expressed, and purified the Galectin-8A gene to investigate its function and to test whether it differs functionally from the Galectin-6 gene.

**Figure 1:**
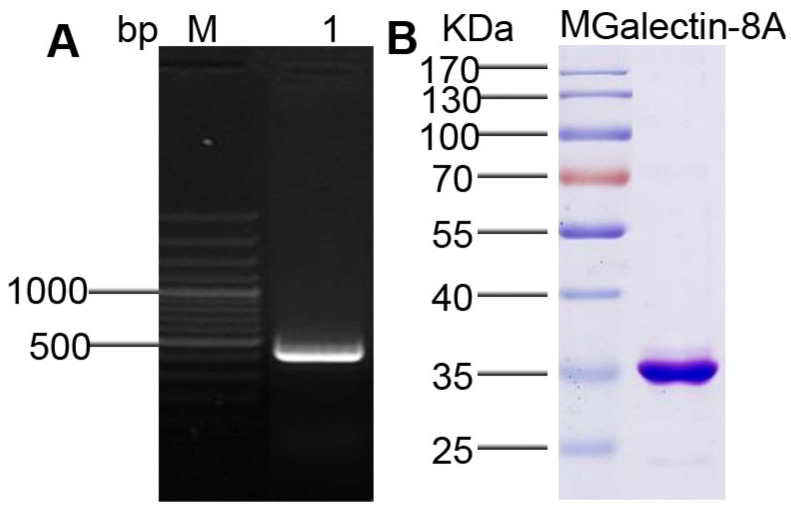
Cloning and recombinant expression of Galectin-8A gene: (**A**) PCR analysis of Galectin-8A gene, lane M, 100 bp plus DNA marker, lane 1 is the PCR product. (**B**) SDS-PAGE analysis of Galectin-8A protein. Lane M is a 10–180 kDa RageRuler protein marker from thermos. Lane Galectin-8A is the purified Galectin-8A thioredoxin fusion protein.

The Galectin-8A gene was amplified by PCR. Based on GenBank accession analyses and assay results, the full-length Galectin-8A gene is about 432 bp (Figure 1A).

In order to express the recombinant Galectin-8A protein, the cloned gene was ligated into a pET32a vector and transformed into a BL21 competent cell. The recombinant Galectin-8A protein was purified using Ni-NTA chromatography. The SDS-PAGE results indicated that the specific protein bands were approximately 35 kDa, which is consistent with the size calculated from the Galectin-8A protein sequence (Figure 1B). The results showed that the Galectin-8A protein was successfully purified.

### 2.2. Galectin-8A Protein Inhibits Larvicidal Activity of Bt Toxins

**Figure 2:**
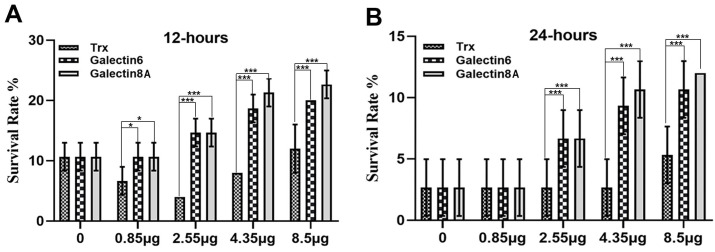
Toxicity bioassay of Bti and the Galectin-8A protein and Galectin-6 protein. *Ae. aegypti* larvae were fed with LLP29 (0.2 μg/mL) mixed with increasing concentrations of Galectin-8A/Galectin-6 thioredoxin fusion protein or thioredoxin (0, 0.85, 2.55, 4.35, or 8.5 μg), and the mosquito larvae survival rate was recorded at 12 h (**A**) and 24 h (**B**) after treatment. Each column represents the mean ± SEM (n ≥3). Significant Trx refers to larvae fed with LLP29 mixed with the pET32a fusion protein. The significance of differences was calculated by one-way ANOVA followed by Tukey’s multiple comparison tests using GraphPad Prism 8.0. Identical letters indicate no significant difference (*p* > 0.05), whereas different letters indicate significant differences (*p* < 0.05).* *p* < 0.05; *** *p* < 0.001.

To determine whether Galectin-8A protein can alter the activity of Bt toxins and whether this effect was consistent with the insecticidal activity of Galectin-6 protein. Third instar larvae of *Ae. aegypti* were fed toxins LLP29 mixed with the purified recombinant Galectin-8A protein or Galectin-6 protein, and their cumulative survival rates were recorded. The toxicity bioassay results showed that the survival rate of *Ae. aegypti* larvae increased when Galectin-8A fusion protein was mixed with LLP29 as compared with control larvae fed on LLP29 mixed with control thioredoxin protein at 12 h and 24 h after feeding (Figure 2). These results suggest that Galectin-8A protein can reduce the larvicidal activity of Bt toxins. According to previous research, Galectin-6 protein suppresses the toxicity of Cry11Aa. We examined whether the Galectin-8A protein was different from the Galectin-6 protein. As shown in Figure 2, the insecticidal activity of the Galectin-8A protein was consistent with that of the Galectin-6 protein, which induces an increase in Bt toxin. However, in the *Ae. aegypti* larvae treated with Bt toxin, the insecticidal activity of the Galectin-8A protein was weaker than that of the Galectin-6 protein. These results indicated that Galectin-8A protein can inhibit the insecticidal activity of Bt toxins, but its inhibitory effect is weaker than that of Galectin-6 protein.

### 2.3. Galectin-8A and Cry11Aa Binds to BBMV

**Figure 3:**
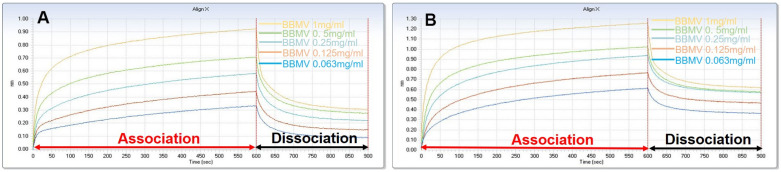
Cry11Aa and Galectin-8A bind to BBMVs of *Ae. aegypti* by Octet Red System. Real-time association and dissociation analysis of Cry11Aa (**A**) and Galectin-8A (**B**) binding to BBMVs. Cry11Aa and Galectin-8A were biotinylated and immobilized to a Streptavidin biosensor, and increasing concentrations of BBMVs from *Ae. aegypti* were run through the coated biosensor.

The key step in the toxicity of Bt toxins is the interaction between the toxins and putative receptors. Cry11Aa has been found to bind to the BBMV of *Ae. aeypti*. To determine whether the Galectin-8A protein could bind to midgut BBMVs, the Octet Red System was used to test the interaction between Galectin-8A or Cry11Aa and BBMVs. Cry11Aa and Galectin-8A were immobilized on SA sensors, and BBMVs were run through the coated sensors. The binding kinetic parameters of Cry11Aa and Galectin-8A to BBMVs were determined by global 1:1 fitting analysis. The results showed that the Kd value for the binding of Cry11Aa to BBMVs was 1.73 × 10^−7^·s^−1^, and Galectin-8A bound to BBMVs was 4.66 × 10^−8^·s^−1^ (Figure 3). These results confirm that Cry11Aa has a weaker affinity for BBMVs than Galectin-8A. As previously reported, the Kd value of Galectin-6 binding to BBMVs was 2.77 × 10^−7^·s^−1^ [23]. The results showed that the Galectin-8A Kd value was smaller than Galectin-6, indicating Galectin-8A has a higher affinity for BBMVs than Galectin-6. This result was consistent with the observation that Galectin-8A reduces the activity of Bt toxins.

### 2.4. Galectin-8A Binds to Individual Toxin Receptors

**Figure 4:**
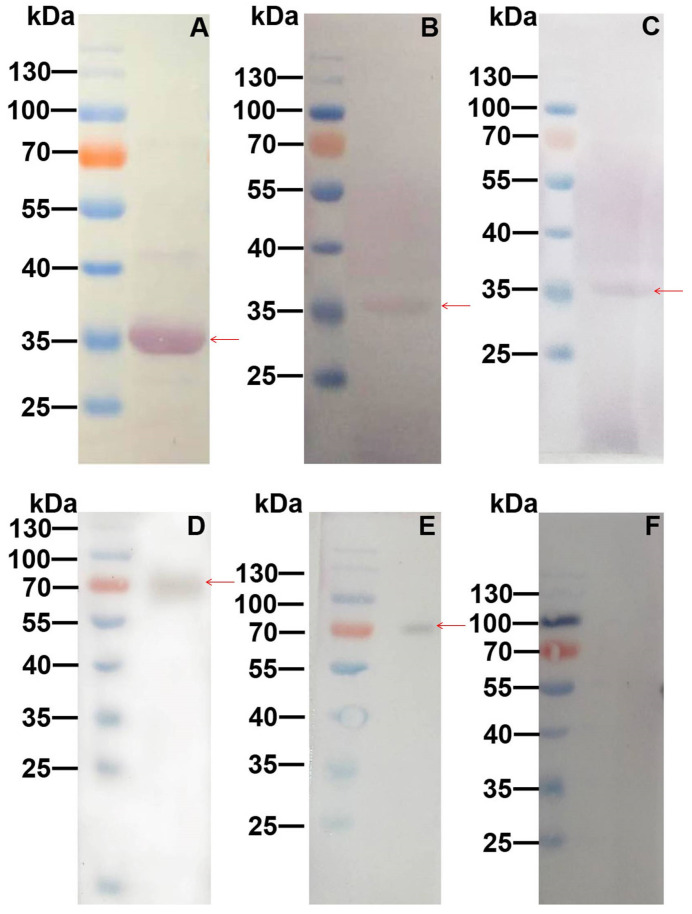
Western blot and far-Western blot analyses of Galectin-8A, ALP1, APN2, and Cadherin. Western blot analysis of the recombinant Galectin-8A protein (**A**) using the Galectin-8A protein antibody. Detection of Galectin-8A bound to ALP1 by far-Western blot after probing with Galectin-8A (**B**). Detection of Galectin-8A bound to APN2 by far-Western blot after probing with Galectin-8A (**C**). Detection of ALP1 bound to Galectin-8A by far-Western blot after probing with ALP1 (**D**). Detection of APN bound to Galectin-8A by far-Western blot after probing with APN2 (**E**). Detection of Cadherin bound to Galectin-8A by far-Western blot after probing with Cadherin (**F**). The red arrow indicates the target band.

Cry11Aa has been found to bind to BBMVs of *Ae. aegypti*, such as ALP, APN, Cadherin, and ATP-binding cassette transporters. To further test whether Galectin-8A binds to individual toxin receptors of *Ae. aegypti*, Western blot and far-Western blot analysis were performed with purified recombinant proteins. The Western blot analysis with the recombinant *Ae. aegypti* Galectin-8A protein showed that the protein band corresponding to Galectin-8A fusion protein was at approximately 35 kDa (Figure 4A). The far-Western blot result showed that the purified recombinant Galectin-8A fusion protein on the membrane was detected by polyclonal rabbit antibody against ALP1 and APN2 when the membrane was probed with ALP1 (~66 kDa) and APN2 (~73 kDa), respectively (Figure 4B,C). Similarly, the ALP1 and APN2 in the membrane were detected by an antibody specific to Galectin-8A after probing with Galectin-8A (Figure 4D,E). However, when Galectin-8A in the membrane was probed with Cadherin, the protein band was not observed after detection with a polyclonal antibody specific to Cadherin (Figure 4F). These results indicate that Galectin-8A could interact with ALP1 and APN2, but not with Cadherin. These results are consistent with previously reported Galectin-6.

**Figure 5:**
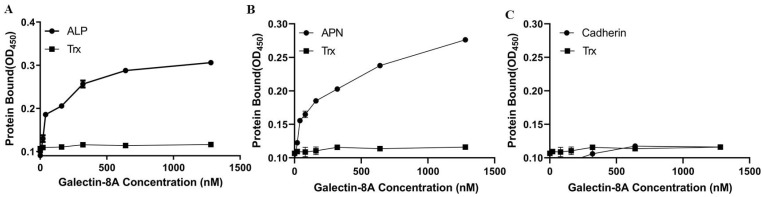
Binding of Galectin-8A to receptor proteins by ELISA. Total binding of biotinylated Galectin-8A and Trx to ALP1 (**A**), APN2 (**B**), and Cadherin (**C**) was determined in the presence of increasing concentrations of biotinylated Galectin-8A and Trx to immobilized ALP1, APN2, or Cadherin.

ELISA assays were conducted to confirm the binding of Galectin-8A to toxin receptors. The results showed that Galectin-8A bound to ALP1 and APN2, but not to Cadherin (Figure 5), which is consistent with the results from the far-Western blot.

### 2.5. Three-Dimensional Protein Structures of Galectin-8A and Molecular Docking

**Figure 6:**
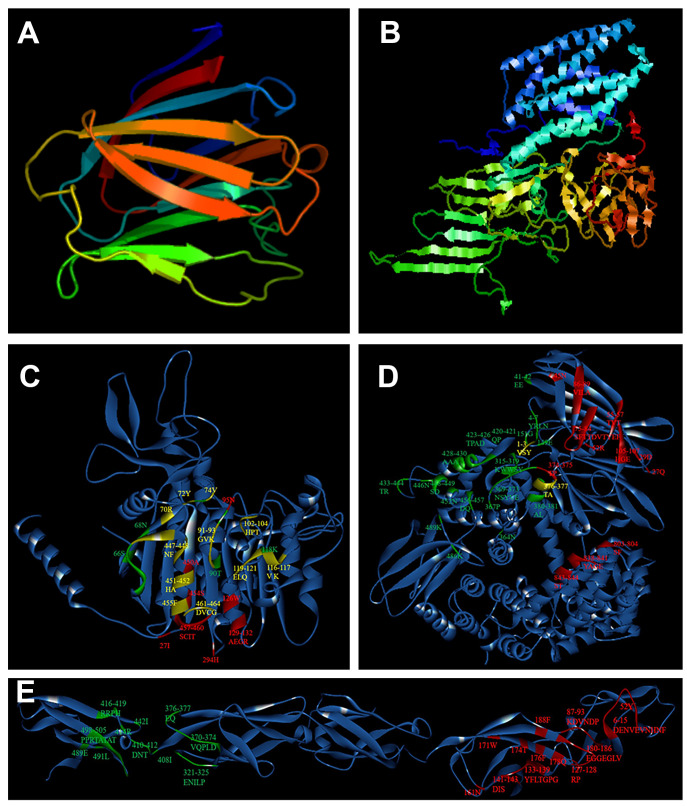
Three-dimensional structure and molecular docking analysis. (**A**): Model structure of Galectin-8A protein. (**B**): Model structure of Cry11Aa. (**C**): Molecular docking of ALP1 with both Galectin-8A and Cry11Aa. (**D**): Molecular docking of APN2 with both Galectin-8A and Cry11Aa. (**E**): Molecular docking of Cadherin with both Galectin-8A and Cry11Aa. The green part is the binding site in the toxin receptors only for Galectin-8A, the red part is the binding sites in the toxin receptors only for Cry11Aa, and the yellow part is the binding sites in the toxin receptors for both Galectin-8 and Cry11Aa.

The online analysis software PHYRE2 was used to model the structure of the Galectin-8A protein. The results revealed that the Galectin-8A model structure is similar to domain II of Cry11Aa (Figure 6A,B). Previous results showed that Galectin-8A and Cry11Aa can both bind to ALP1 and APN2, though we would like to know whether Galectin-8A and Cry11Aa bind to the same regions in ALP1 or APN2; thus, molecular docking of ALP1, APN2, or Cadherin with both Galectin-8A and Cry11Aa was performed. The results showed that Galectin-8A was docked to ALP1, APN2, and Cadherin. When ALP1 or APN2 was docked with Galectin-8A, amino acids in the receptor were identified at the interface of the three proteins, especially in the case of ALP1. However, this was not detected among Galectin-8A and Cry11Aa docked with Cadherin (Figure 6E). These results indicate that the positions of these amino acids at the interface of the three proteins may be important binding sites in the receptors for the Galectin-8A protein. In addition, compared with the Galectin-6 binding to ALP1 and APN2 [21], the results showed that the Galectin-8A protein binding sites were different from Galectin-6. Therefore, different binding sites may lead to different binding affinities with toxins. These results indicate that different Galectin proteins bind to Cry toxin at different regions, resulting in different biological activities. This study may provide an interesting perspective regarding the role of galectins in the mechanism of Bt toxins and an opportunity for the development of new biological pesticides for mosquito control.

## 3. Discussion

Bt can produce crystal proteins such as Cry4Aa, Cry4Ba, Cry10, Cry11Aa, and Cyt1Aa, which were found to be toxic to mosquitoes. Among them, Cry11Aa has proven to be one of the most active mosquitocidal agents against *Ae. aegypti*. Cry11Aa has been found to bind to BBMVs of *Ae. aegypti*, such as ALP, APN, Cadherin, and ATP-binding cassette transporters. Previously reported studies indicated that Cry toxin has a three-domain structure, with domain II primarily responsible for binding to the receptor through recognition of the anchored glycosyl groups on glycoprotein, and it has a β-prism structure similar to that of galectins.

Galectins are a family of β-galactoside-binding proteins that are widely distributed in insect species and play an important role in insect immune response. In our previous work, we found that five galectin genes were expressed in the midgut of *Ae. aegypti* [18]. Bioassays showed that Galectin-6 can reduce the toxicity of Cry11Aa, thereby protecting *Ae. aegypti* larvae. At the same time Galectin-6 can compete with Cry11Aa for binding to the receptor ALP1 and APN2, resulting in a significant increase in the survival of mosquito larvae after being treated with Cry toxins [22]. Otherwise, it is not known whether other galectins in the midgut of *Ae. aegypti* was related to Bt insecticidal activity. In this study, the Galectin-8A protein reduced the toxicity of Cry11Aa, consistent with the Galectin-6 protein. However, Galectin-8A protein showed an increased inhibitory effect on toxins than Galectin-6 protein. At the same time, using the Octet Red System to detect the Galectin-8A protein interaction with *Ae. aegypti* BBMVs. The results showed that Galectin-8A can interact with BBMVs, and the Kd value is smaller than that of Galectin-6 protein. This indicates that Galectin-8A has stronger interactions with BBMV than Galectin-6 protein. The Western blot and far-Western blot analysis showed that Galectin-8A is able to bind ALP1 and APN2 receptors but not Cadherin. Similarly to Galectin-6, the amino acids in the ALP1 and APN2 are important for binding to Cry11Aa, and therefore may be the key residues for competitive binding. In order to detect the Galectin-8A protein bind to receptor activity, molecular docking predicted that residues in Galectin-8A were involved in competition with Cry11Aa for binding to the midgut receptors protein. Moreover, the Galectin-8A binding sites were different from Galectin-6. Thus, different binding sites can lead to different binding abilities with toxins. This study may provide an interesting perspective regarding the role of galectins in the mechanism of Bt and an opportunity for the development of new biological pesticides for mosquito control.

## 4. Conclusions

This study cloned, expressed, and purified the recombinant protein of Galectin 8 protein. The bioassay results indicated that Galectin-8A is similar to Galectin-6 in reducing the toxicity of Cry11Aa. But the Galectin-8A is stronger in inhibiting toxins than Galectin-6. In order to determine interactions among Galectin-8A, Cry11Aa, and toxin receptors, Octet Red System, Western blot, and far-Western blot assay were also performed. The Octet Red System result showed that Galectin-8A can also bind to BBMVs of *Ae. aegypti*. The Western blot and far-Western blot indicated that Galectin-8A can block Cry11Aa from binding to ALP1, APN2, which might decrease the mosquitocidal activity of the toxin. But the Octet Red System kDa value is smaller than that of Galectin-6, indicating that Galectin-8A had a stronger binding ability to BBMVs than Galectin-6. This study may provide an interesting perspective regarding the role of galectins in the mechanism of Bt and an opportunity for the development of new biological pesticides for mosquito control.

## 5. Materials and Methods

### 5.1. Mosquitoes and Bacterial Strains

*Ae. aegypti* (Haikou strain) were provided by the Fujian International Travel Health Care Center (Fuzhou, China). The mosquitoes were reared in an environmentally controlled room at 28 °C and 85% RH with a photoperiod of 14 h light and 10 h dark. Bti LLP29 was isolated from *Magnolia denudate* [32]. The Cry11Aa was provided by Dr. Sarjeet Gill, University of California, Riverside.

### 5.2. Expression and Purification of Recombinant Galectin-8A

*Ae. aegypti* Galectin-8A (GenBank accession no. AAEL005293) was amplified by polymerase chain reaction using gene-specific primers (Forward primer: 5-CATGCCATGGCCCAACTGCCAGTG-3; Reverse primer: 5-CCCAAGCTTTCACTCCATCACGATGGC-3). PCR product was ligated into pMD18T (TaKaRa, Dalian, China) and transformed into *Escherichia coli* JM109. The sequences were verified by Sangon Biotechnology (Shanghai, China). After confirmation, the DNA fragment was ligated into the pET32a expression vector and transformed into *E. coli* BL21.

To express recombinant Galectin-8A protein, the strain was cultivated in LB liquid medium with 0.1% *Amp* at 37 °C for 2 h and then induced with 1mM isopropyl-β-D-1-thiogalactopyranoside (IPTG) at 16 °C for 12 h. The Galectin-8A recombinant protein was purified using Ni Sepharose 6 FF (GE Healthcare, Boston, MA, USA). Galectin-8A polyclonal antibodies were produced by Beyotime (Shanghai, China).

### 5.3. Bioassays

The half lethal concentration (LC_50_) of the LLP29 protein to the third instar larvae of *Ae. Aegypti* was determined. According to the preliminary bioassay results, LLP29 total toxins (0.2 μg/mL) mixed with different concentrations of the recombinant Galectin-8A protein (0, 0.85, 2.55, 4.35, or 8.5 μg) [18]. Each concentration gradient was repeated 3 times. After 12 to 24 h of toxin treatment, the mortality of *Ae. aegypti* larvae were recorded. Further, results were analyzed by the GraphPad Prism software 8.0, and each bar represents the mean ± SD of three replicates.

### 5.4. Preparation of Ae. aegypti Brush Border Membrane Vesicles (BBMVs)

Brush border membrane vesicles (BBMVs) of Ae.aegypti larvae were isolated according to the previously published method of Abdul-Ruaf 1999 assay [33]. Briefly, the midgut of the fourth instar *Ae. aegypti* larvae were isolated, resuspended in MET buffer A (0.3 M mannitol, 5 mM EGTA, 17 mM Tris-HCl) containing 0.1% (*w*/*v*) protein inhibitor (Roche), and fully ground on ice with a homogenizer. An equal volume of 24 mmol/l MgCl_2_ solution was added, allowed to stand on ice for 15 min, and then centrifuged at 2500× *g* at 4 °C for 15 min. Separate the supernatant and then add 500 μL of MET buffer, ground again, followed by adding an equal volume of MgCl_2_ kept on ice for 15 min, and centrifuged at 30,000× *g*, at 4 °C for 30 min. The resulting pellet was resuspended in 50 μL MET buffer. The BBMV protein concentration was measured using the Bradford assay.

### 5.5. Western and Far-Western Blot Analysis

The recombinant *Ae. aegypti* ALP1, APN2, and Cadherin were purified as reported [26,27].

For the Western blot analysis, the purified Galectin-8A, Cry11Aa, ALP, APN2, and Cadherin proteins were separated on 10% SDS-PAGE and then transferred to nitrocellulose membranes. The membrane was blocked with 5% dry skim milk in PBS containing 0.05% Tween-20 (PBST) and incubated with primary rabbit polyclonal antibody (1:2000) to each protein for 2 h, washing with PBST three times. The secondary antibodies (1:5000) reacted with membrane-bound primary antibodies to each protein for 2 h, after washing with PBST three times. Binding of specific antibody was detected by BCIP/NBT Alkaline Phosphatase Color Development Kit (Beyotime, Shanghai, China) according to the manufacturer’s instructions.

### 5.6. Octet Red System

The Octet red system is the surface interference technology of the biofilm layer. It was used to accurately detect the affinity of Cry11Aa with BBMV or Galectin-8A. Octet red system was carried out following the manufacturer’s instructions (Thermo Fisher Scientific, Waltham, MA, USA). The purified Cry11Aa and Galectin-8A proteins were biotinylated with EZ-Link-NHS-Biotin and then immobilized on streptavidin biosensors, and increasing concentrations (63, 125, 250, 500, and 1000 nM) of BBMVs were run through the protein-coated biosensors. The dissociation rate (Kd) and relative dissociation constant for the binding of Cry11Aa or Galectin-8A to BBMVs were analyzed using the Octet red 96 system Data Analysis software.

### 5.7. Elisa

To further investigate the interaction between Galectin-8A binding of different putative toxin receptors was assessed using a modified microplate assay. Briefly, 96-well plates were coated with 4 μg/well of ALP1, APN2, Cadherin, and incubated overnight at 4 °C, and then washed three times with PBS. Increasing concentrations of biotinylated Galectin-8A (0 to 1280 nm) or the control thioredoxin (0 to 1280 nm) in 100 μL of PBST (0.1% Tween 20, 1×PBS, pH 7.4) were then added to the coated plates. After 2 h, the plates were washed three times with 100 μL/well of PBST. Bound biotinylated proteins were detected by incubation of the plates with streptavidin–horseradish peroxidase (HRP) conjugate (1:3000) for 1.5 h, and assayed for HRP using TMB Horseradish Peroxidase Color Development Solution for ELISA (Beyotime, Shanghai, China). Enzyme activity was determined based on the rate of increase in optical density at 450 nm.

### 5.8. Modeling and Protein Docking Analyses of Galectin-8A, Cry11Aa, ALP, APN, and Cadherin

The three-dimensional protein structures of Cry11Aa (PDB:IDLC), ALP1 (PDB:IK7H), APN2 (PDB:4WZ9), and Cadherin (PDB:4UX8) were obtained from the online analysis software PHYRE2.2 (http://www.sbg.bio.ic.ac.uk/phyre2/html/page.cgi?id=index) accessed on 29 April 2018 [21]. The human Galectin-8 (PDB: 4FQZ) was used as the template for Galectin-8A. Protein docking was conducted with the Discovery Studio 2.5. software. The docking complexes were obtained using the GRAMM-X Protein–Protein Docking Web Server v.1.2.0 (http://vakser.compbio.ku.edu/resources/gramm/grammx) accessed on 29 April 2018 and analyzed through the mean square deviation of the receptor and binding interface.

## Data Availability

The original contributions presented in this study are included in the article. Further inquiries can be directed to the corresponding author.
